# The Haiti Medical Education Project: development and analysis of a competency based continuing medical education course in Haiti through distance learning

**DOI:** 10.1186/s12909-016-0795-x

**Published:** 2016-10-19

**Authors:** Robert Battat, Marc Jhonson, Lorne Wiseblatt, Cruff Renard, Laura Habib, Manouchka Normil, Brian Remillard, Timothy F. Brewer, Galit Sacajiu

**Affiliations:** 1 Department of Gastroenterology, McGill University Health Centre (MUHC) , 1001 Decarie Blvd, D05-7161, Montreal, H4A 3J1 Canada; 2 Service Medecine Interne, Hopital Universite d’Etat d’Haiti, Port-au-Prince, Haiti; 3 Department of Medicine, McGill University, Montreal, Canada; 4 Department of Hospital Medicine, St Elizabeth Medical Center, Edgewood, KY USA; 5 Department of Internal medicine, McGill University, Montreal, Canada; 6 Department of Family Medicine, Hopital St. Nicolas, St Marc, Haiti; 7 Dartmouth Hitchcock Medical Center, One Medical Center Drive, Lebanon, NH USA; 8 Department of Medicine, David Geffen School of Medicine, University of California Los Angeles, Los Angeles, USA; 9 ARC Health Resources, Congers, NY USA

**Keywords:** Global health, Medical education, Distance learning

## Abstract

**Background:**

Recent calls for reform in healthcare training emphasize using competency-based curricula and information technology-empowered learning. Continuing Medical Education programs are essential in maintaining physician accreditation. Haitian physicians have expressed a lack access to these activities. The Haiti Medical Education Project works in alliance with Haitian medical leadership, faculty and students to support the Country’s medical education system. We present the creation, delivery and evaluation of a competency-based continuing medical education curriculum for physicians in rural Haiti.

**Methods:**

Real time lectures from local and international institutions were teleconferenced to physicians in remote Haitian sites using VidyoConferencing™ technology. With American Academy of Family Physicians (AAFP) and College of Family Physicians Canada (CFPC) guidelines as references, a competency-derived syllabus was created for a Haitian continuing medical education program. The resulting educational goals were reviewed by a committee of Haitian and North American physician/medical education practitioners to reflect local needs. All authors reviewed lectures and then conferred to establish agreement on competencies presented for each lecture.

**Results:**

Sixty-seven lectures were delivered. Human immunodeficiency virus/Acquired Immunodeficiency Syndrome, ophthalmologic, infectious diseases, renal and endocrine competencies were well-represented, with more than 50 % of the joint AAFP and CFPC recommended competencies outlined. Areas under-represented included allergy and immunology, cardiology, surgery, pain management, gastroenterology, neurology, pulmonology, men’s health and rheumatology; these topics accounted for less than 25 % of AAFP/CFPC recommended competencies. Areas not covered included geriatrics, nutrition, occupational health and women’s health. Within practice-based lectures, only disaster medicine, health promotion and information management were included, but only partially covered.

**Conclusions:**

We identified teaching goals covered and competencies that were missing from a CME program for rural Haitian physicians. We aim to use this analysis to provide a competency-based CME lecture series that proportionally meets local needs while following recommendations of recognized national family medicine organizations.

## Background

To meet the goals set by the Universal Declaration of Human Rights to promote health and ensure adequate access to medical care [1], health systems need to insure that health care providers are well-trained and competent [2]. Recent international calls for major reform in healthcare professional training have emphasized using competency-based curricula and information technology-empowered learning [3]. Competency-based curricula have become the preferred means of delivering medical education [3–5]. Unfortunately, those countries with the greatest healthcare needs often have the fewest educational resources to advance health care provider training [6].

Advanced communication and interactive distance learning tools provide unique opportunities to bring innovative educational resources to medical professionals in low-income or remote locations, thereby expanding global access to high-quality training programs [3, 7, 8]. Besides enabling access to educational materials and instructors, distance learning programs can assist with supporting relationships among medical professionals across distant locations and provide valuable opportunities for capacity building [9].

Continuing medical education (CME) has long been used in high-income settings to facilitate the ongoing acquisition of knowledge and skills by health care professionals with the aim of improving patient care [10]. Despite the important role CME programs have in maintaining physician accreditation in high-income countries, there currently have been few opportunities for ongoing CME in low-income countries [11, 12].

The Republic of Haiti, which occupies the western third of the Island of Hispaniola, is one of the poorest countries in the Americas [13]. Haitian health indices are the lowest ranking in the Western Hemisphere and amongst the lowest in the world [14–17]. Despite a long tradition of medical education in Haiti, medical education resources remain inadequate to meet the country’s needs [14–18]. Haitian physicians have expressed a lack access to professional development programs and CME activities [6]. The Haiti Medical Education (HME) Project, a non-profit organization, works in alliance with Haitian medical leadership, faculty and students to support the Country’s medical education system by bringing together healthcare providers, academics and social activists across multiple countries to work towards restoring and building upon the infrastructure and curricula of Haitian medical schools and teaching hospitals [19–23].

One area of active effort for HME and its Haitian and international partners has been the establishment of CME opportunities for practicing physicians. Currently, there is no published literature addressing competency based curricula delivery through distance learning in developing countries. We present the creation, delivery and evaluation of the early stages of a competency-based CME curriculum for physicians working in rural Haiti using videoconferencing technologies to provide a series of lectures from locally and internationally-based experts and supported by local academic clinical programs.

## Methods

### Distance learning tools

Prior to the initiation of this lecture series in April 2011, individual hospitals held local teaching session, but no multi-site lecture series existed at rural sites. In order to reach Haitian physicians based at remote rural sites, VidyoConferencing™ technology was used for teleconferencing lectures in real time from international institutions to the training locations. High-quality audiovisual lectures were provided to multiple sites, despite limited Internet connectivity. Participants could ask questions directly to the instructor or to colleagues across the linked sites during the presentation. Lectures were delivered pro-bono by academically affiliated experts from Haiti or abroad.

### Establishing competencies

Lectures initially were given based on lecturer availability without predetermined competency-based educational objectives. To provide more relevant and targeted CME materials for our audience, a competency-derived CME curriculum was created, with the aim of structuring our course with pre-determined competencies. This process included analyzing the presentations that had been given to date to determine those competencies that had been delivered and those that had not.

**Fig. 1:**
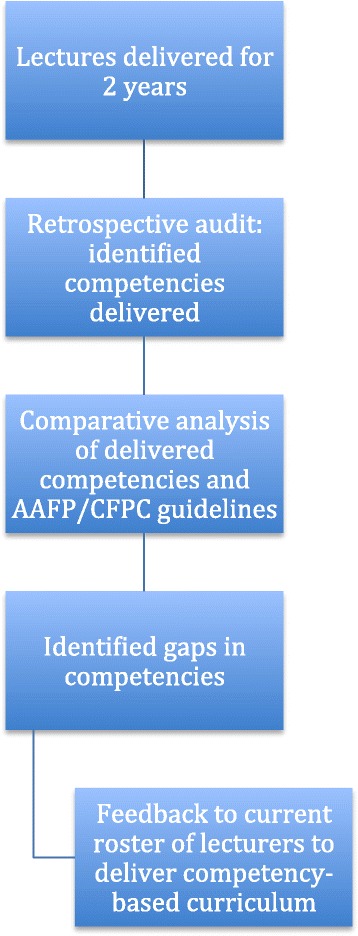
Strategy for Identifying Competencies Delivered

Using the American Academy of Family Physicians (AAFP) and College of Family Physicians Canada (CFPC) guidelines as references [24, 25] a competency-derived syllabus was created for a Haitian continuing medical education program. AAFP and CFPC competencies were categorized by topic area (e.g., cardiology, infectious disease, public health, etc.), and then 38 AAFP defined competency areas were abstracted and re-organized to create a baseline set of lecture topics and learning objectives. This initial set was then cross-referenced using the CFPC competency guidelines to identify additional areas for inclusion. The resulting educational goals were reviewed by a committee of Haitian and North American physician/medical education practitioners (RB, CR, LW, MJ, MN, TFB and GS) to ensure that the final set of competency objectives were appropriate for local Haitian practice needs (Fig. 1). Forty-four competency domains, broadly divided into “disease-based competencies” and “practice-based competencies” for organizational purposes were created (http://www.hmeproject.org/competencyexcel/). For the purposes of this program, a competency was defined as “the habitual and judicious use of communication, knowledge, technical skills, clinical reasoning, emotions, values, and reflection in daily practice for the benefit of the individual and the community being served” [26].

### Audit of delivered competencies

To assess the coverage of competency-based learning objectives, each lecture was analyzed individually and separately by two reviewers (RB, CR, LW, MJ, MN, TFB or GS), who were blinded to the other’s evaluation. This analysis was performed for of recordings of online video-lectures delivered from April 1st 2011 to January 1st 2013. Competencies delivered in these lectures were identified using the established AAFP/CFPC competency syllabus. Furthermore, delivered competencies were then cross-referenced with this syllabus. Competencies that were expected based on the presentation topic but not covered in the lecture also were noted. The reviewers then conferred to establish agreement on competencies presented for each reviewed lecture. In the cases where the 2 reviewers did not reach consensus, a separate senior reviewer (GS) adjudicated discrepancies.

## Results

Sixty-seven lectures were delivered between April 1st 2011 and January 1st 2013. Lectures delivered, lecturer information and affiliations are outlined in Table 1. Sixty-three lectures addressed disease-based competencies and 4 lectures addressed practice-based competencies. Competencies delivered were compared to the established AAFP/CFPC competency syllabus (Table 2).

**Table 1:** Lectures and Competency Areas Delivered

Competency area	Physician lecturer name	Lecturer affiliation	Lecture title:
Cardiology	Martin Sedlacek	Dartmouth University	Hypertension-Blood Pressure
	Juan Carlos Chirgwin	McGill University	(1) Hypertension - Risk Factors, Screening, Investigations (2) Hypertension - Therapy
	Jocelyne David	University of Miami	(1) Heart Failure (2) Pre-operative evaluation
	Joseph Valery Andre	St. Vincent Medical Center	Secondary Hypertension: Etiology, Diagnosis and Management
	Brian Remillard	Dartmouth University	(1) Hypertension (2) Hypertensive Emergencies
Endocrinology	Kaberi Dasgupta	McGill University	Management of Type II Diabetes
	David Morris	McGill University	Thyroid Disease
Urgent and Emergent Care	Robert Harris	Dartmouth University	Abdominal Emergencies with Ultrasound
	Robert Hyde	Dartmouth University	Anaphylaxis
	Joseph Valery Andre	St. Vincent Medical Center	The Recognition and Management of Shock States
	Dickens Saint-Vil	Université de Montreal	Prise en charge des traumas pédiatriques
	Thomas Joseph Lydon	Wentworth-Douglass Hospital	Therapeutic Hypothermia
	Jacques Stanley Elysse	Laval University	Prise en charge des AVC ischmiques
Family Medicine	Pierre Paul-Tellier	McGill University	Men’s Health
Hematology	Cruff Renard	Albert Einstein College of Medicine	(1) Anemia (2) Sickle Cell Anemia
Infectious Diseases	Tim Brewer	McGill University	Cellulitis
	Selim Rashed	Université de Montreal	(1) Malaria (2) Giardiasis and Amebiasis (3) Dengue (4) Giardiasis and Filiariasis
	Marie- Josée Brouillette	McGill University	Cognitive disorders associated with HIV infection and their impact on the treatment of HIV
	Makeda Semret	McGill University	Acute Meningitis
	Jodie Dionne-Odom	Dartmouth University	(1) HIV PEP/PreP (2) STIs - Gonorrhea, Chlamydia, Syphilis and HPV
	Mark Wainberg	McGill University	(1) Pharmacoresistance to HIV (2) Understanding HIV Drug Resistance
	Pierre Paul-Tellier	McGill University	Sexually Transmitted Infections
Nephrology	Brian Remillard	Dartmouth University	Acute Renal Failure
	Michelle Elizov	McGill University	Acute Kidney Injury
	Serge Quérin	Université de Montreal	Dysnatrémies et dyskaliémies: approche pratique
	Brian Remillard	Dartmouth University	L’hemodialyse de suppleance
	Martin Sedlacek	Dartmouth University	Urinalysis and its use as a diagnostic and management measure
Obstetrics and Gynecology	David Morris	McGill University	Diabetes in Pregnancy
	Thomas Lydon	Wentworth-Douglass Hospital	First-Trimester Vaginal Bleeding
	Roger Duvivier	Albert Einstein College of Medicine	(1) Prevention of Maternal Mortality (2) La mortalité maternelle en Haiti
	Daniel Blouin	Université de Sherbrooke	Porblemes Obstetricaux
Oncology	Thierry Alcindor	McGill University	(1) Colon Cancer (2) Chimiothérapie-Principes de base
	Robert Fine	Columbia University	Pancreatic Cancer
Opthalmology	Franz Large	Haitian Society of Opthalmology	What every doctor must know about Opthalmology
Orthopedics	Marc Rambaud	Centre hospitalier de Sens, France	(1) Orthopedic Issues & Case Discussion (2) Les fractures bi-Malléolaires, Orthopedic Issues & Case Discussion
Other	Galit Sacajiu	Albert Einstein College Of Medicine	Reading the Literature - Clinical Epidemiology Workshop
	Pierre Minn	University of California, San Francisco	La Recherche Qualitative
	Alison Doucet	McGill University	Pain Management
	Pierre Paul-Tellier	McGill University	Testicular Mass
	Thomas Minde	McGill University	Médecine Corps-Ėsprit (Médecine Psychosomatique)
Pediatrics	Pia Wintermark	McGill University	(1) Common Neonatal Brain Injuries (2) Management of a newborn with hypoxic-ischemic encephalopathy (3) Management of Low-Birth Weight Infants
	Harris Huberman	State University of New York	Autistic Spectrum Disorder and its identification and management as it might apply in the context of Haiti
	Andrea Gorgos	McGill University	Pediatric follow-up of premature baby
	Shuvo Ghosh	McGill University	Language Development in Children
Psychiatry	Katlyne Lubin	Albert Einstein College Of Medicine	Cognitive-Adaptive Abilities
	Marc Laporta	McGill University	Mental Health post-Emergency/Natural Disaster
Respirology	Ron Olivenstein	McGill University	(1) Asthma: Guidelines for Diagnosis and Therapy (2) COPD/Emphysema
Rheumatology	Marshall Fleurant	Boston University	(1) Shoulder Pain (2) Back Pain - Outpatient Management and Diagnosis
	Marc Rambaud	Centre hospitalier de Sens, France	(1) Arthrose du genou, (2) La hanche de l’enfant (the hip of the child)
	Lucie Brazeau	Université de Sherbrooke	Quelques indices sur les RX de l’épaule, utiles au raisonnement clinique á propos de l’épaule

**Table 2:** Evaluation of Competencies Deliverered

Disease based lectures					
Competency area	Topic areas (% of AAFP/CFPC competencies)		For each topic area	Lectures (n)	Total lectures (%)
Adolescent		Overlap with Other Topics		0	
Allergy	14 %		67 %	2	3 %
Cardiology	21 %		89 %	7	10 %
Infants and Childern	31 %		100 %	6	9 %
Older Adults		Not Covered		0	0 %
Critical Care		Overlap with Other Topics		1	1 %
Surgical	7 %		56 %	2	3 %
Pain	13 %		44 %	1	1 %
Endo	53 %		89 %	3	4 %
Eye	52 %		67 %	3	4 %
GI	20 %		44 %	1	1 %
Neuro	10 %		100 %	2	3 %
Resp	16 %		89 %	2	3 %
Heme	38 %		67 %	2	3 %
HIV/AIDS	100 %		N/A	3	4 %
Human Behaviour	55 %		78 %	3	4 %
ID	79 %		100 %	7	10 %
Mat and Gyne	36 %		33 %	5	7 %
Mens Health	15 %		0 %	2	3 %
Muscoloskeletal	44 %		100 %	2	3 %
Nutrition		Not Covered		0	0 %
Occupational		Not Covered		0	0 %
Renal	59 %		78 %	3	4 %
Rheum	8 %		91 %	2	3 %
Emerg	31 %		100 %	4	6 %
Womens Health		Not Covered		0	0 %
Practice Based Lectures					
Disaster Medicine	6 %		N/A	1	1 %
Global Health		Not Covered		0	0 %
Graduate Medical Education		Not Covered		0	0 %
Health Promotion	19 %		N/A	1	1 %
Info Management	91 %		N/A	2	3 %
Leadership		Not Covered		0	0 %
Management of Health Systems		Not Covered		0	0 %
Medical Ethics		Not Covered		0	0 %
Medical Informatics		Not Covered		0	0 %
Office Laboratory		Not Covered		0	0 %
Patient Education		Not Covered		0	0 %
Practice Based Learning		Not Covered		0	0 %
Risk Management and Medical Liability		Not Covered		0	0 %
Urban Practice Curriculum		Not Covered		0	0 %

Within disease based lectures, human immunodeficiency virus/Acquired Immunodeficiency Syndrome (HIV/AIDS), ophthalmologic, general infectious diseases, renal and endocrine competencies were well-represented; together these subjects covered more than 50 % of the joint AAFP and CFPC recommended competencies outlined. Recommended competency areas under-represented in the lecture series included allergy and immunology, cardiology, surgery, pain management, gastroenterology, neurology, pulmonology, men’s health and rheumatology; together these topics accounted for less than 25 % of AAFP/CFPC recommended competencies. Recommended competency areas not covered included geriatrics, nutrition, occupational health and women’s health. In general, when a specific topic area was covered by a lecture, the majority of recommended joint AAFP/CFPC learning goals for that topic area were presented. Within practice-based lectures, only disaster medicine, health promotion and information management were included in lectures; however, the competency goals for these topics were only partially covered. Recommended practice-based topics not covered by lecturers are listed in Table 2.

A full outline of competencies covered, by competency area, is available on HME website (http://www.hmeproject.org/competencyanalysis/).

## Conclusions

Competency-based medical education is useful for initiating and maintaining targeted continuing medical education learning. A collaboration between Haitian and North American physicians led to a live distance learning CME program available to physicians in rural Haiti delivered via video conferencing technology. Using an educational syllabus jointly derived from AAFP and CFPC competencies for family physicians, 67 lectures were provided covering greater than 50 % of AAFP and CFPC recommended competencies. Within each topic area presented, lecturers succeeded in covering specific teaching points completely.

Having the syllabus was valuable in identifying several competency areas recommended for family physicians but that were underrepresented in the lectures. Examples of topics not covered by lectures by recommended by the guidelines included acute coronary syndromes, management of weight loss, breast diseases and meningitis. While lecturer availability was the primary constraint for deciding which topics were presented, finding ways to present the range of recommended topics will be important in the future to ensure that participants are exposed to the gamut of topic areas needed to be a well-rounded family physician.

Expanding presentations of the practice-oriented competencies also needs to be done; for example, only mental health in disasters was discussed among the recommended disaster management topics. Skill-based competencies, such as Advanced Cardiac or Trauma Life Support, also were not included. As the program develops, establishing skill-based competencies using distance learning technologies will be a major challenge to be overcome.

Finally, it is notable that no global health topics such as burden of disease, migration and travel, social determinants of health, or health as a human right were discussed. While global health topics are expanding in North American undergraduate and graduate medical education, these topics also are increasingly important for postgraduate medical education in other high-income and middle/low-income regions [5, 27, 28]. Global health root cause analysis with an emphasis on social justice is already underway in Haiti [29]; however, continued education and widening the audience for this topic would be valuable.

This analysis demonstrated the value of having competency-based curriculum to identify teaching goals covered and competencies that were missing from a CME program for rural Haitian physicians run over approximately 18 months. We aim to use this analysis to target future lecturer recruitment to provide a competency-based CME lecture series that proportionally meets local needs while following recommendations of recognized national family medicine organizations. Further, with a competency based lecture series, curriculum evaluations can be performed. An ancillary benefit of this collaboration has been the bi-directional learning for all participants involved in this partnership. Lecturers not only offer a service through providing high-quality lectures, but they also gain important experience through participating in long-term partnerships with Haitian colleagues. This lecture series presents an innovative approach to delivering CME to low-income country primary care providers in remote areas that could be adapted by for other locations.

## Abbreviations

AAFP: Academy of Family Physicians
CFPC: College of Family Physicians Canada
CME: Continuing Medical Education
HME Project: Haiti Medical Education Project
